# Synthesis of Telechelic‐Type Polypeptides Functionalized with Aromatic Units and the Characterization of Their Structures and Thermal Properties

**DOI:** 10.1002/marc.202500499

**Published:** 2025-08-26

**Authors:** Yusuke Ueno, Kousuke Tsuchiya, Hiroyasu Masunaga, Keiji Numata

**Affiliations:** ^1^ Department of Material Chemistry Graduate School of Engineering Kyoto University Kyoto Japan; ^2^ Department of Chemistry and Biotechnology Graduate School of Engineering The University of Tokyo Tokyo Japan; ^3^ Japan Synchrotron Radiation Research Institute Hyogo Japan; ^4^ Biomacromolecules Research Team RIKEN Center for Sustainable Resource Science Saitama Japan

**Keywords:** chemoenzymatic polymerization, polypeptide, self‐assemblies, telechelic polymers, thermal stability, *π*–*π* stacking

## Abstract

To enhance their physical and thermal properties, telechelic polypeptides with extended peptide chains from aromatic diamines were synthesized via the chemoenzymatic polymerization of diamine‐type initiators and amino acid esters. With the introduction of meta‐substituted aromatic diamines, atomic force microscopy (AFM) revealed the formation of large aggregates in telechelic polyalanine (TPA), which was attributed to *π*‒*π* stacking interactions between aromatic rings, as well as short fibrous aggregates with branched structures. The introduction of rigid aromatic rings also improved the thermal stability of the polypeptides.

## Graphical abstract

Polypeptide chains were extended from both ends of aromatic diamines by chemoenzymatic polymerization, resulting in the synthesis of telechelic polyglycines (TPG) and polyalanines (TPA). These telechelic polymers assembled into branched nanofibrils through *π*‐*π* stacking of aromatic rings, with the polymer backbones bent at the aromatic unit. The incorporation of aromatic rings also imparted enhanced thermal stability.

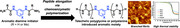

## 1 Introduction

The unique properties of natural polymeric materials arise from the hierarchical structures of biopolymers synthesized by specific organisms. By understanding these hierarchical architectures and elucidating the mechanisms underlying their physical properties, it becomes possible to artificially replicate the distinctive functionalities of natural polymers and biopolymers [1]. Polypeptides have a wide range of applications as extremely useful materials because of the diverse structures and properties expressed by various amino acid sequences. For example, polyalanine generally self‐assembles through intermolecular hydrogen bonds to form antiparallel β‐sheets. This β‐sheet structure is known to exist as crystalline domains in various proteins. In silk proteins such as spider silk, crystalline regions, including polyalanine motifs, contribute to high tensile strength [2, 3]. In addition to silk proteins, structural proteins such as elastin, collagen, and resilin also exhibit specific mechanical properties through highly repetitive amino acid sequences in proteins. Elastin and resilin have high elasticity and mechanical strength [4, 5], whereas collagen has excellent mechanical strength [6]. If polypeptides based on these protein motifs can be chemically synthesized, it will be possible to develop and produce functional materials of biological origin on a large scale.

There are many reports on the chemical synthesis of polypeptides that mimic these structural proteins [7, 8], allowing artificial expression of the unique properties and functionalities of these proteins by designing appropriate sequences. Polypeptide synthesis methods include solid‐phase peptide synthesis (SPPS) and ring‐opening polymerization of the amino acid *N*‐carboxyanhydride (NCA). SPPS allows precise control of amino acid sequences but requires multiple synthetic steps, such as repeated condensation and deprotection. Therefore, SPPS is unsuitable for synthesizing long‐chain peptides. In contrast, NCA polymerization can produce peptides with relatively large molecular weights, but it primarily results in the formation of random or block copolymers in multicomponent polymerization, limiting precise sequence control. Chemoenzymatic polymerization, which utilizes the reverse reaction of protease‐mediated hydrolysis, is characterized by superior atom efficiency and low environmental impact due to its ability to polymerize in aqueous solvents [9]. Additionally, the synthesis of defined dipeptides or tripeptides as monomers prior to chemoenzymatic polymerization enables the efficient introduction of precise repeating sequences into polypeptides [10, 11, 12].

The physical properties and self‐assembled structures of polypeptides strongly depend on the primary structure, including the amino acid sequence and polymer chain length. Introducing unnatural structures into the main chain enables the development of specific structures and properties that are not achievable with natural peptides [13]. For example, incorporating aromatic rings into the main chain leads to a unique secondary structure via *π*‒*π* stacking of the benzene rings, resulting in thermoplasticity and improved thermal stability due to the rigid structure of the aromatic ring‐incorporated backbone [14]. In addition to introducing periodic unnatural structures, propagating peptide chains from multifunctional building blocks leads to nanostructures that differ from homopolymers, thereby imparting unique properties not found in natural peptides. Telechelic polypeptides, in which two peptide chains extend in opposite directions from the unnatural moiety, assemble into unique fibrous nanostructures distinct from linear homopolymers [15]. Utilizing this feature of fibrous aggregation, the toughness of composite materials (films and fibers) with silk fibroin has been reported to be greater than that of silk alone or homopolymers [16, 17]. Mixing the telechelic polypeptides also enhanced the mechanical properties of synthetic polymer matrices such as poly(vinyl alcohol) [18]. The fabrication of shape‐memory materials by synthesizing a copolymer consisting of telechelic polyalanine as a crystalline building block and poly(ε‐caprolactone) as a soft block mediated with hexamethylene diisocyanate has also been reported [19].

On the basis of these previous studies, we hypothesize that synthesizing a telechelic polypeptide with an aromatic diamine incorporated at the middle junction between two polypeptide chains will result in unique self‐assembled structures, which are expected to enhance physical properties such as high thermal stability, owing to the *π*‒*π* interactions of rigid aromatic moieties. In this study, telechelic polypeptides were synthesized via chemoenzymatic polymerization in the presence of an initiator consisting of an aromatic diamine moiety to enhance the physical properties of natural homopolypeptides due to their specific self‐assembly. In particular, the use of meta‐substituted aromatic rings is expected to induce more specific self‐assembled structures by altering the molecular geometry from a linear to a bent conformation. The structure, morphology, and thermal properties of the synthesized polymers were evaluated, and the effects of the introduction of aromatic rings were discussed. Telechelic polymers are expected to serve as useful building blocks for block copolymers and may be applicable to the synthesis of peptide‐based functional polymers because of their tendency to be self‐assembled.

## 2 Results and Discussion

### 2.1 Synthesis of Telechelic Polymers

**SCHEME 1:**
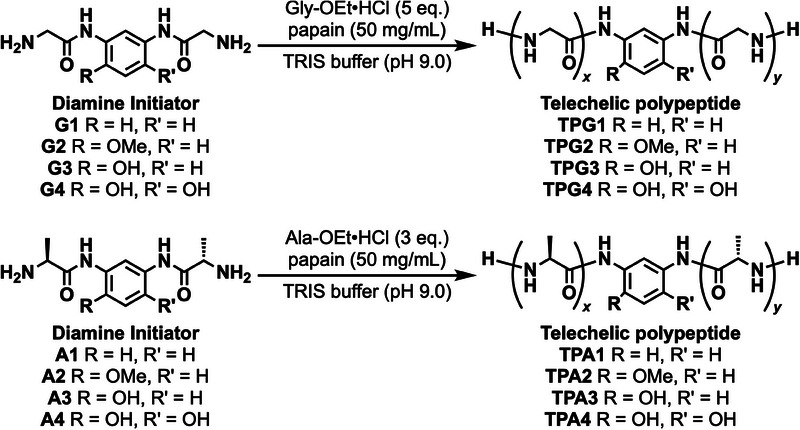
Synthesis of telechelic polypeptides via chemoenzymatic polymerization.

When unnatural structures are introduced into the main chain of polypeptides by chemoenzymatic polymerization via papain, these unnatural units are rarely recognized by the substrate recognition site of papain. Therefore, previous examples of enzymatic polymerization have employed a molecular design in which an unnatural moiety is sandwiched between natural amino acids such as glycine and alanine to increase enzyme recognition [13, 14]. Since aromatic diamines are generally not recognized by papain, we decided to use glycine and alanine to modify the aromatic moieties at the two amino groups for initiators. The diamine initiators **G1‐4** and **A1‐4**, synthesized by condensing meta‐substituted aromatic diamines with glycine or alanine via liquid‐phase synthesis, were used for papain‐catalyzed polymerization (Scheme 1). Polymerization was carried out via the addition of 5 and 3 equivalents of glycine ethyl ester or alanine ethyl ester to each initiator, respectively, in tris(hydroxymethyl)aminomethane (Tris) buffer at 40°C for 6 h. Since the alanine‐sandwich initiator exhibited lower enzyme recognition than did the glycine‐sandwich initiator, we sought to suppress the formation of the polyalanine homopolymer by reducing the molar equivalents of alanine relative to the glycine‐based system. For all the initiator/monomer combinations, the corresponding telechelic polypeptides were obtained as a precipitate after the polymerization.

**FIGURE 1:**
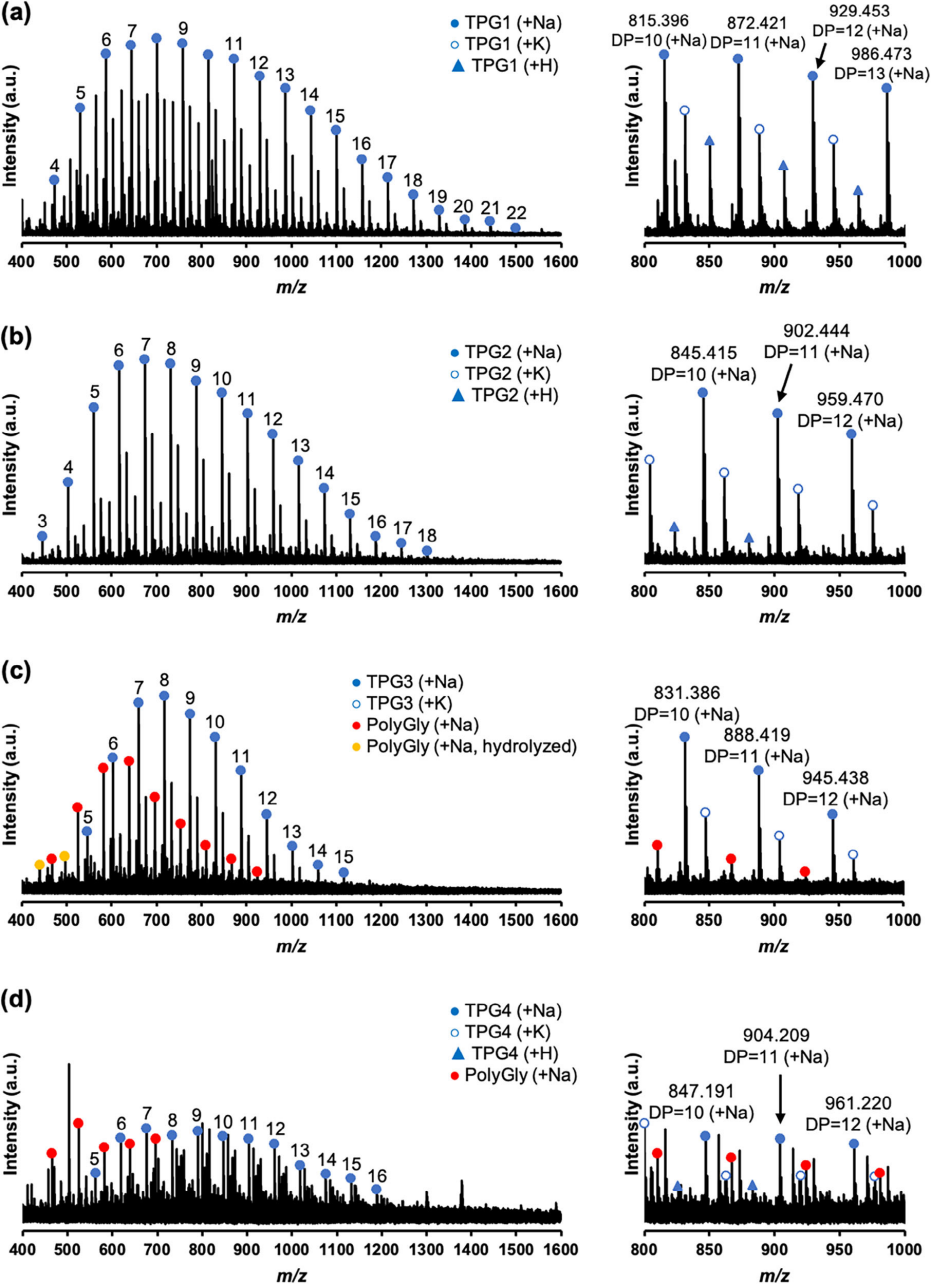
MALDI‐TOF MS spectra of (a) **TPG1**, (b) **TPG2**, (c) **TPG3**, and (d) **TPG4** (matrix:α‐CHCA).

**FIGURE 2:**
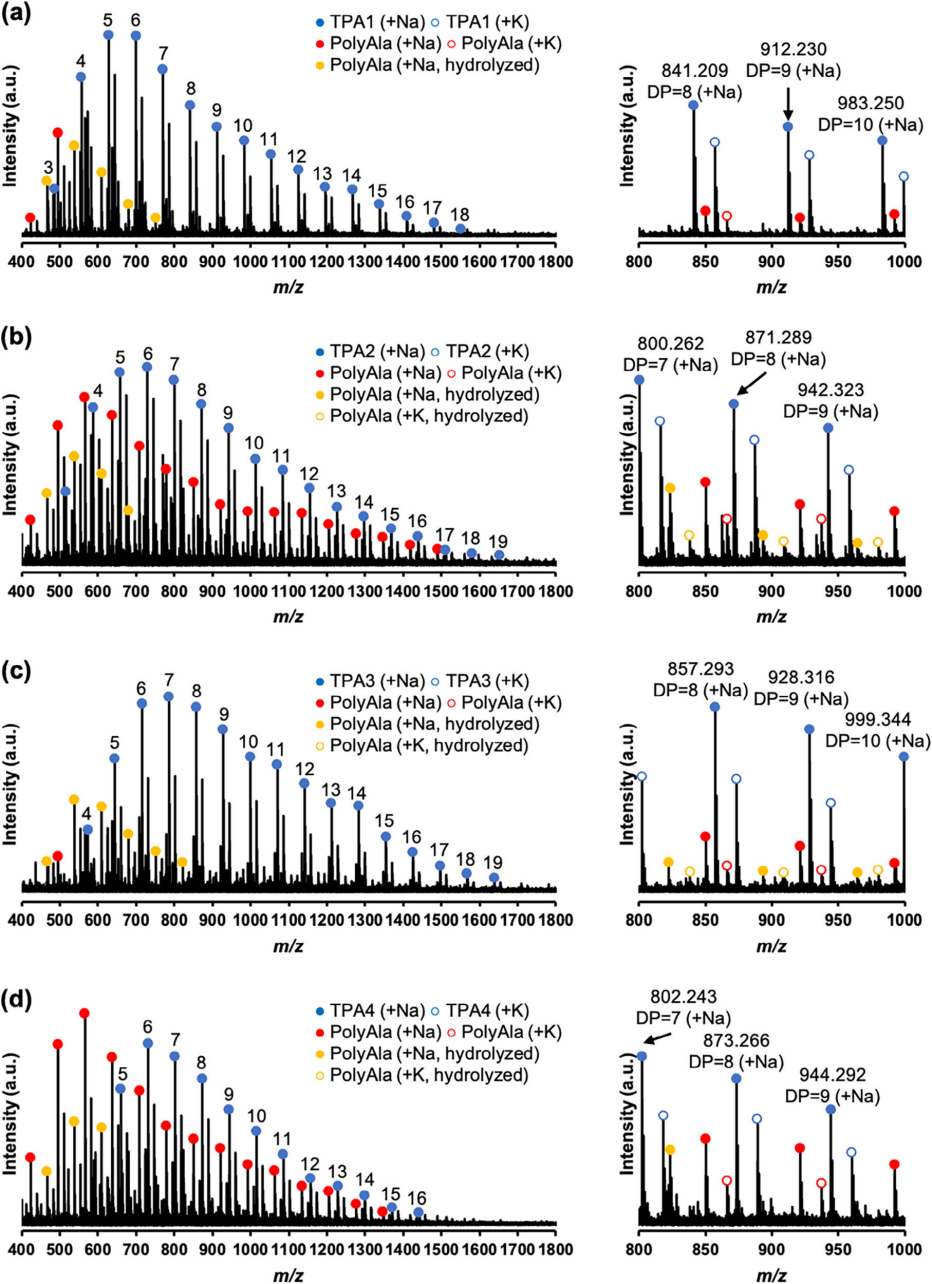
MALDI‐TOF MS spectra of (a) **TPA1**, (b) **TPA2**, (c) **TPA3**, and (d) **TPA4** (matrix:α‐CHCA).

As shown in Table 1, the average degree of polymerization (DP) for telechelic polyglycine (TPG) ranged from 8.7 to 13.4, with the maximum DP observed between 15 and 22. Analysis by matrix‐assisted laser desorption/ionization time‐of‐flight mass spectrometry (MALDI‐TOF MS) (Figure 1) confirmed the formation of polyglycine elongated from aromatic diamine initiators, with a peak interval of molecular mass corresponding to 57 *m*/*z* of the glycine residue. However, minor series of peaks corresponding to glycine homopolymers (red or yellow circles in Figure 1c,d) were also observed for **TPG3** and **TPG4**. For telechelic polyalanine (TPA), the formation of polyalanine elongated from aromatic diamines was observed, with an average DP ranging from 10.7 to 14.0 and a maximum DP ranging from 16 to 19. The MALDI‐TOF MS spectra (Figure 2) revealed a series of peaks corresponding to alanine homopolymers (red or yellow circles in Figure 2) in all the systems. As shown in Table 1, the formation of telechelic polymers was limited to 40% to 60%, particularly for **TPA3** and **TPA4**. Similar to the glycine case, the insolubility of **A3** and **A4** in the Tris buffer resulted in the majority of the homopolypeptide precipitates.

**TABLE 1:** Chemoenzymatic synthesis of **TPG1‐4** and **TPA1‐4**.

Polymer	Yield^a^ (%)	DPmax^b^	DP^c^	TPG/TPA content^c^ (%)
**TPG1**	10.8	22	11.4	>99
**TPG2**	4.4	18	13.7	>99
**TPG3**	17.7	15	12.1	>99
**TPG4**	20.2	16	8.7	34.6
**TPA1**	14.1	18	11.4	59.2
**TPA2**	17.6	18	11.6	50.9
**TPA3**	17.9	19	14.0	48.7
**TPA4**	24.5	16	10.7	41.2

^a^ Precipitate was collected via centrifugation, washed with water, and lyophilized.

^b^ Determined by MALDI‐TOF MS.

^c^ Determined by ^1^H NMR.

### 2.2 Structural Characterization

The structures of **TPG1‐4** and **TPA1‐4** were characterized by 1H nuclear magnetic resonance (NMR), Fourier transform infrared (FT–IR), and wide‐angle X‐ray diffraction (WAXD) analyses. In the 1H NMR spectra of **TPG1‐4** (Figure S1), a peak corresponding to the α‐protons of glycine was observed at 4.3 ppm, and a peak corresponding to the protons of the aromatic ring appeared at 7.5–6.5 ppm. No homopolymer formation was observed for **TPG1‐3**, while the formation of the polyglycine homopolymer was confirmed in **TPG4** by the peaks corresponding to the terminal ethyl ester at 4.6 and 1.2 ppm. The 1H NMR spectra of **TPA1‐4** (Figure S2) also showed peaks corresponding to the α‐proton and α‐methyl protons of alanine at 4.3 and 1.2 ppm, respectively. Small peaks corresponding to the protons of the aromatic ring were observed between 7.5 and 6.5 ppm, indicating the formation of telechelic polyalanine with the aromatic initiator unit. However, as mentioned earlier, the peaks corresponding to the terminal ethyl ester of the polyalanine homopolymer were observed for all the TPAs with higher contents than TPG, indicating the competitive formation of the polyalanine homopolymer.

**FIGURE 3:**
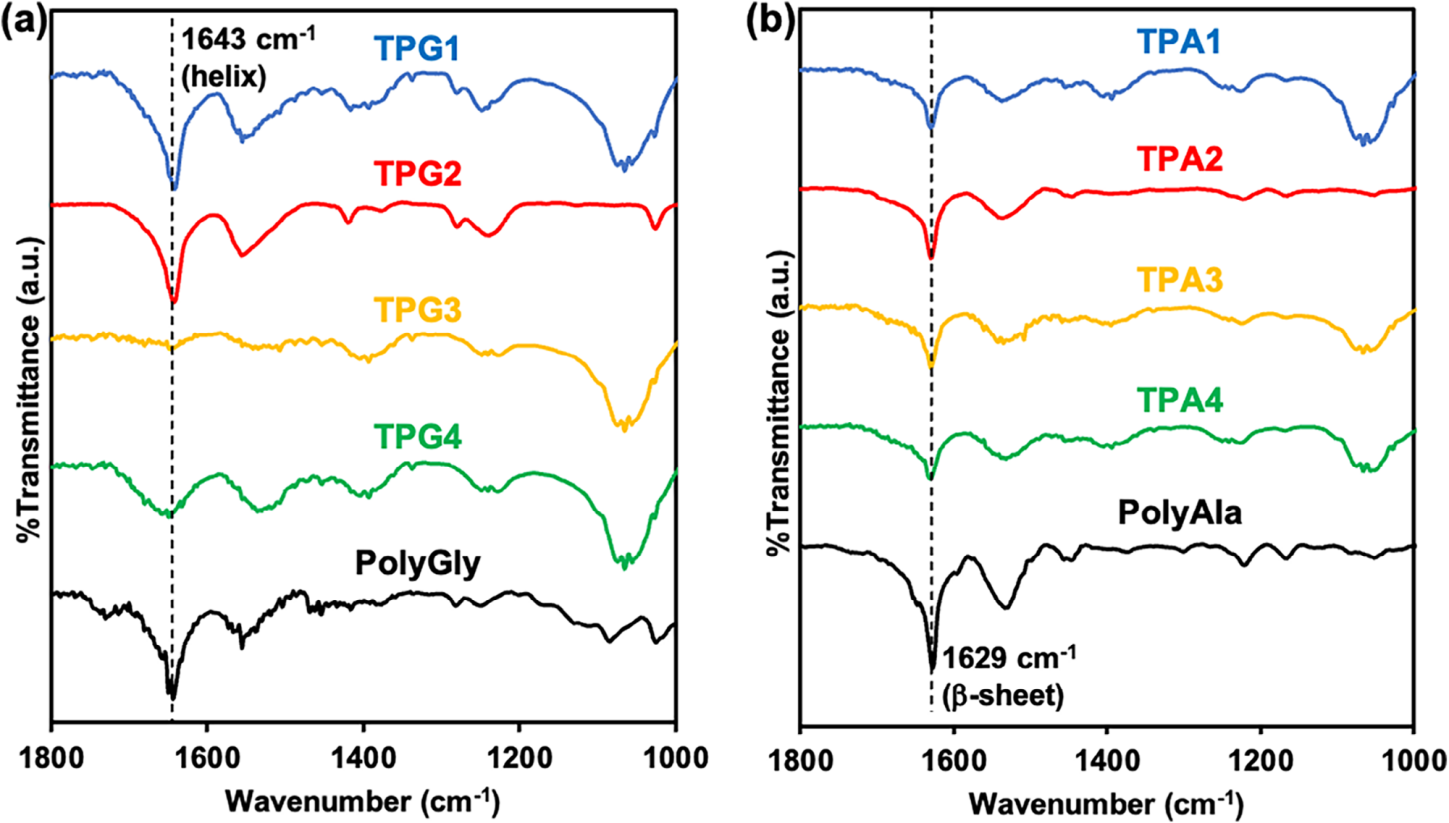
FT–IR spectra of (a) **TPG1‐4** and PolyGly and (b) **TPA1‐4** and PolyAla.

To evaluate the secondary structure of these polymers, FT‐IR was performed. The FT‐IR spectra revealed a sharp amide I peak at 1643 cm^−1^ for the TPGs and at 1629 cm^−1^ for the TPAs (Figure 3). For comparison, homopolymers of glycine (PolyGly) and alanine (PolyAla) were measured, and the same sharp peaks were observed. This suggests that the telechelic polypeptides have a secondary structure similar to that of the homopolymers, a threefold helix of polyglycine II [20] for TPGs and a β‐sheet [21] for TPAs. However, no peak corresponding to the initiator aromatic ring was observed, likely because the structure of the peptide chain extends in both directions from the aromatic diamine and the small proportion of aromatic rings in the polymer from the aromatic diamine, and because of the small proportion of aromatic rings in the polymer.

**FIGURE 4:**
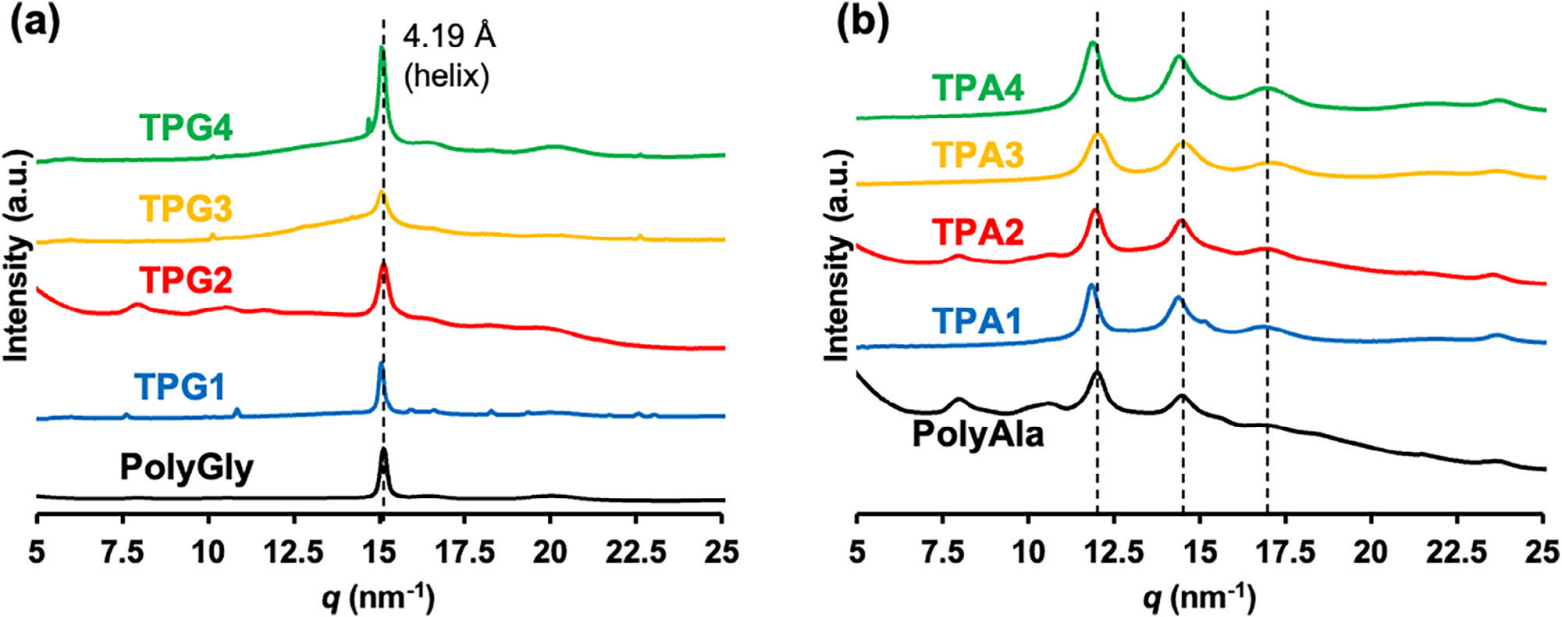
WAXD profiles of (a) **TPG1‐4** and PolyGly, (b) **TPA1‐4** and PolyAla.

To investigate the secondary structures in more detail, the crystalline structures of the polymer powder were analyzed via WAXD (Figure 4). A peak corresponding to an interplanar spacing of 4.19 Å was observed for all the TPG polymers. This corresponds to the distance between the polymer chains in the hexagonal close‐packed structure of polyglycine II, which forms a three‐helix structure [22]. This suggests that a secondary structure similar to that of PolyGly was obtained for the TPGs. The full width at half maximum (FWHM) of the peak at *q* = 15.2 nm^−1^ for all TPGs was broader than that of PolyGly (0.25–0.44 nm^−1^), confirming the peak broadening in TPG compared with PolyGly (Table S1). This suggests that the introduction of an aromatic ring at the middle junction disrupts the regularity of the polymer arrangement, resulting in smaller crystallite sizes. For TPAs, three characteristic peaks of 5.23, 4.33, and 3.67 Å interplanar spacings were observed, which correspond to the antiparallel β‐sheet crystalline structure of polyAla,[23] indicating that TPAs adopt an antiparallel β‐sheet structure similar to that of polyAla. The FWHM of the peak at 3.67 Å, corresponding to the (020) plane of the β‐sheet, was relatively broader than that of PolyAla (Table S2). The (020) plane of the polyAla β‐sheet corresponds to the distance between the sheets, and a slight shift to a lower *q* value suggests that the distance between the sheet planes increased. This may be due to the disruption of the β‐sheet structure of polyAla by *π*–*π* stacking at the aromatic ring. The relative increase in the intensity of the peaks corresponding to other β‐sheet‐forming planes ((210) and (211) planes) also suggested that the introduction of aromatic rings into the middle junction of the main chain disrupted the regularity of the polyAla arrangement.

### 2.3 AFM Observations

Polyalanine is known to self‐assemble into nanoarchitectures through intermolecular interactions between amide bonds, forming a β‐sheet structure. It has also been reported that elongation of the polymer chain in polyAla leads to the formation of fibrillar aggregates [24]. Previous studies have shown that telechelic polyAla tends to form fibrillar aggregates at lower molecular weights than polyAla of similar molecular weights [15]. Aromatic polyamides, on the other hand, tend to form highly planar self‐assemblies through *π*‒*π* stacking of aromatic rings, leading to increased rigidity due to a high degree of crystallization. Therefore, we hypothesize that the telechelic polyalanine with aromatic diamines at the middle junction in this study can also form fiber‐like self‐assemblies while generating sheet‐like aggregates with enhanced planarity.

To assess the morphology of the synthesized crystalline aggregates of **TPA1‐4**, atomic force microscopy (AFM) was carried out on the **TPA1‐4** aggregates deposited on mica substrates. In this study, the synthesized **TPA1‐4** was dispersed in a 1:1 (v/v) mixture of hexafluoro‐2‐propanol (HFIP) and methanol at a concentration of 1 mg mL^−1^, deposited onto a mica substrate, and subsequently dried. As a control, linear polyAla without aromatic rings was evaluated under the same conditions.

**FIGURE 5:**
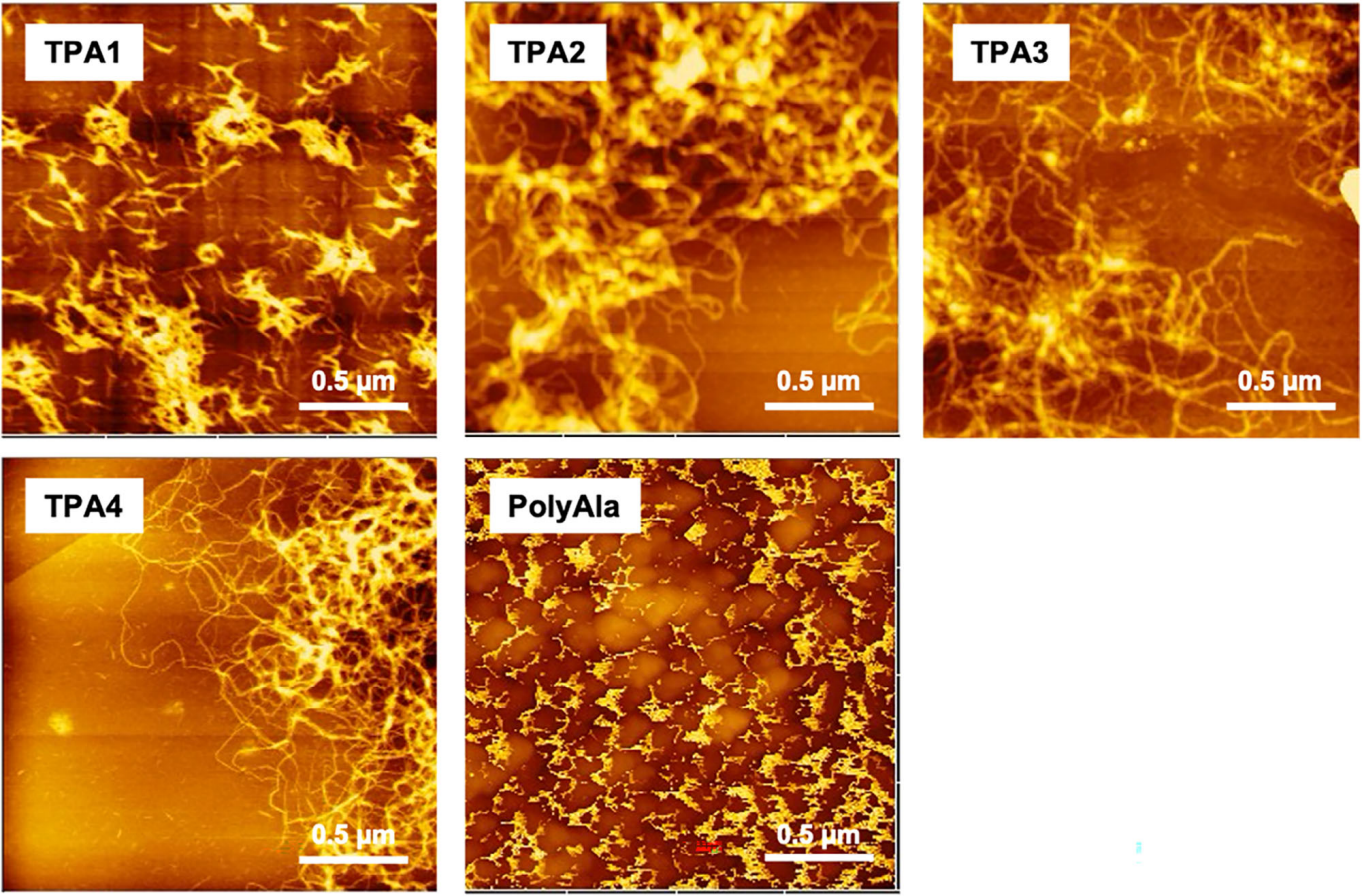
AFM topographic images of **TPA1‐4** and PolyAla deposited on a mica substrate.

**FIGURE 6:**
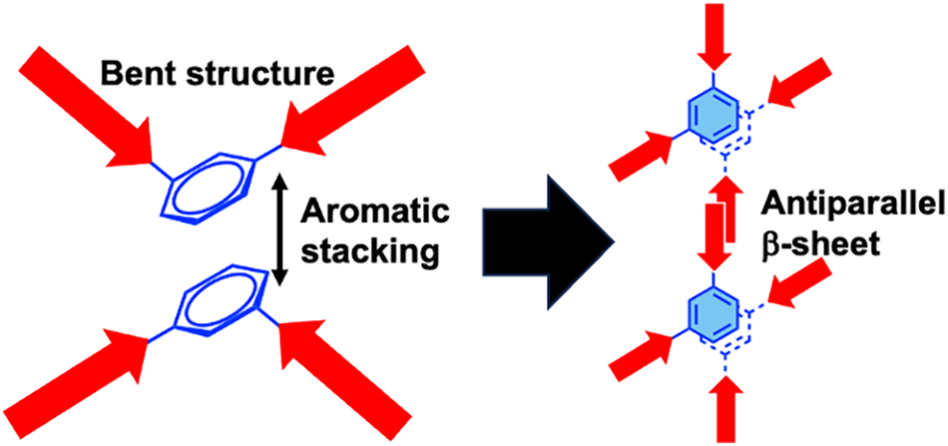
Conceptual illustration of the self‐assembly behavior of TPAs with an aromatic unit.

Representative AFM images of **TPA1‐4** and polyAla are shown in Figure 5. Granular aggregates were observed for linear polyAla, whereas short fibrous aggregates with branched structures were consistently detected for **TPA1‐4**. A previous study reported that TPAs synthesized from initiators based on aliphatic linear dicarboxylic acids formed long, fiber‐like aggregates spanning several micrometers [15, 24, 25]. In contrast, the TPAs described in this report possess rigid meta‐substituted aromatic diamines at the middle junction between the polyalanine segments, which leads to a distinct molecular structure bent around the aromatic ring. Consequently, the bent structure resulting from intermolecular interactions via *π*‒*π* stacking of the aromatic rings putatively induces critical morphological differences from previous TPAs containing aliphatic initiator units, resulting in unique aggregates with a branched, networked structure (Figure 6). Notably, the branched/networked structure was more clearly observed for **TPA4**, indicating that the introduction of hydroxy groups at the aromatic moiety facilitated molecular assembly into the branched fibrous morphology.

### 2.4 Thermal Properties

Polypeptides with aromatic rings in the main chain are reported to have improved thermal stability because of their rigid structure [26]. We evaluated the thermal stability of the telechelic polypeptides via thermogravimetric analysis (TGA) (Figure S3). We found that **TPG1‐3** presented greater thermal stability than did the homopolymer PolyGly, whereas **TPG4** presented a weight loss of approximately 100°C due to the evaporation of water. No weight loss due to water evaporation was observed in **TPGs 1–3**. In contrast, **TPG4** exhibited greater moisture absorption than the other TPG samples and was not completely dry, suggesting that the observed weight loss was attributable to water evaporation. For the TPAs, the weight loss after 350°C was smaller than that of the homopolymer polyAla because of the incorporation of robust aromatic rings. However, this change was less significant than that of the TPGs. Considering that the 5% and 10% degradation temperatures were lower than those of polyAla, the facilitated degradation might be due to a disruption of the secondary structure of polyalanine caused by the introduction of aromatic rings.

The results were also compared with previously synthesized telechelic polyglycine (aliphatic‐TPG) [15] and polyalanine (aliphatic‐TPA) [16] extended from aliphatic dicarboxylic acids (Figure S4). The degradation temperature of TPG synthesized in this study was higher than that of aliphatic‐TPG, which may be attributed to the enhanced thermal stability of the aromatic main chain compared with its aliphatic counterpart. In contrast, the decomposition temperature of TPA did not differ significantly from that of aliphatic‐TPA, suggesting that the introduction of nonnatural structures may reduce the thermal stability by disrupting the secondary structure.

The introduction of unnatural structures into polypeptides has been reported to confer thermoplastic properties that are absent in polypeptides composed solely of amino acids [14, 27], and DSC analysis was performed to investigate the thermoplastic properties (Figure S5). Similar to those of the homopolymers, no melting or glass transition below the decomposition temperature was observed for either of the telechelic polymers, indicating a lack of thermoplasticity. The high occupancy of polyglycine and polyalanine moieties in the polymer structure may explain the minimal impact of the relatively low content of the aromatic ring. Due to the poor thermal processability of TPG and TPA, we could not perform further thermal and rheological analysis. However, the excellent thermal properties of TPG and TPA would be beneficial for practical applications to composite formulation with other polymer materials to enhance the physical properties of the composites, as demonstrated previously [16, 17, 18].

## 3 Conclusion

Chemoenzymatic polymerization was employed to synthesize telechelic‐type polypeptides with extended peptide chains from initiators **G1‐4** and **A1‐4** via the use of aromatic diamine derivatives. The use of an aromatic diamine with glycine or alanine condensed at both ends as an initiator enhanced recognition by papain, enabling the synthesis of telechelic polypeptides. The secondary structures of TPG and TPA were similar to those of the homopolymers, but the introduction of aromatic rings into the main chain disrupted the regularity of the polymer arrangement, indicating the amorphous properties of TPG and a disrupted β‐sheet structure in TPA. TPA adopts a β‐sheet structure similar to that of linear polyalanine, but the crystalline aggregates are suggested to form short fibrous structures branching from the aromatic ring moiety. These telechelic polypeptides can be considered unique building blocks in the synthesis and development of various functional materials. In particular, TPA can be applied as a hard block in thermoplastic elastomers because of the high crystallinity of the β‐sheet structure. Additionally, the facile formation of self‐assemblies through the stacking of aromatic rings may lead to the development of new hierarchical structures. With respect to thermal stability, a slower weight loss than that of the homopolymers was observed because of the robust structure of the aromatic rings. This tendency was particularly pronounced for the TPG. The physical properties and characteristics of TPG and TPA reported in this study are expected to enhance composites with other polymers, including silk, by imparting improved stiffness and thermal performance. These materials have the potential to serve as bio‐based alternatives with high functionality and properties comparable to those of bulk materials, such as commercially available polyamides.

## 4 Experimental Procedure

### 4.1 General Procedure for the Chemoenzymatic Synthesis of TPG1‐4

**G1** (0.40 mmol, 118.1 mg), Gly‐OEt∙HCl (2.0 mmol, 279.2 mg) and 1 m tris(hydroxymethyl)aminomethane (Tris) buffer (3 mL, pH 9.0) were added to a 10‐mL glass tube equipped with a stir bar, and the mixture was stirred at 40°C until all the substrates were completely dissolved. The monomer mixture was poured into a glass tube equipped with a magnetic stirrer, set in a reaction device (ChemiStation, AYELA, Tokyo, Japan) at 40°C, and stirred at 800 rpm. Papain powder (150 mg, 50 mg mL^−1^) was then added to the monomer solution to start polymerization. The mixture was stirred at 40°C and 800 rpm for 6 h. After cooling to room temperature, the precipitate was collected by centrifugation at 9000 rpm and 4°C for 15 min. The crude product was washed three times with deionized water and lyophilized to afford a white TPG1 powder. The yield was 21.9 mg (10.8%). The chemical structure of the product was characterized by 1H NMR spectroscopy and MALDI‐TOF MS. **TPG2**, **TPG3,** and **TPG4** were also synthesized via the same experimental procedure.

### 4.2 General Procedure for the Chemoenzymatic Synthesis of TPA1‐4

**A1** (0.50 mmol, 161.6 mg), Ala‐OEt∙HCl (1.5 mmol, 230.4 mg) and 1 m tris(hydroxymethyl)aminomethane (Tris) buffer (3 mL, pH 9.0) were added to a 10‐mL glass tube equipped with a stir bar, and the mixture was stirred at 40°C until all the substrates were completely dissolved. The monomer mixture was poured into a glass tube equipped with a magnetic stirrer, set in a reaction device (ChemiStation, AYELA, Tokyo, Japan) at 40°C, and stirred at 800 rpm. Papain powder (150 mg, 50 mg mL^−1^) was then added to the monomer solution to start polymerization. The mixture was stirred at 40°C and 800 rpm for 6 h. After cooling to room temperature, the precipitate was collected by centrifugation at 9000 rpm and 4°C for 15 min. The crude product was washed three times with deionized water and lyophilized to afford a white TPA1 powder. The yield was 42.6 mg (14.1%). The chemical structure of the product was characterized by 1H NMR spectroscopy and MALDI‐TOF MS. **TPA2**, **TPA3,** and **TPA4** were also synthesized via the same experimental procedure.

## Conflicts of Interest

The authors declare no conflicts of interest.

## Supporting information




**Supporting File 1**: marc70045‐sup‐0001‐SuppMat.docx.

**Supporting File 2**: marc70045‐sup‐0002‐FigureS1.png.

**Supporting File 3**: marc70045‐sup‐0003‐FigureS2.png.

**Supporting File 4**: marc70045‐sup‐0004‐FigureS3.png.

**Supporting File 5**: marc70045‐sup‐0005‐FigureS4.png.

**Supporting File 6**: marc70045‐sup‐0006‐FigureS5.png.

## Data Availability

The data that support the findings of this study are available in the supplementary material of this article.
