# Supplementary material for: Synthesis of Telechelic‐Type Polypeptides Functionalized with Aromatic Units and the Characterization of Their Structures and Thermal Properties
Source: Macromol Rapid Commun. 2025 Aug 26;47(10):e00499. doi: 10.1002/marc.202500499 (PMC13193375; doi:10.1002/marc.202500499)
Supplement: Supplementary file 1 — Supporting File 1: marc70045‐sup‐0001‐SuppMat.docx. [file MARC-47-e00499-s005.docx]

Supporting Information

# Synthesis of telechelic-type polypeptides functionalized with aromatic units and the characterization of their structures and thermal properties

*Yusuke Ueno, Kousuke Tsuchiya***, Hiroyasu Masunaga, Keiji Numata**

**Experimental procedure**

**Materials**

Papain was purchased from Merck Millipore (Burlington, Massachusetts, United States) and used as received (EC number: 3.4.22.2). The activity was approximately 30000 USP units/mg, where one unit was defined and adopted by the United States Pharmacopeia. 1-(3-Dimethylaminopropyl)-3-ethylcarbodiimide hydrochloride (EDC∙HCl) and ethyl cyano(hydroxyimino) acetate (OxymaPure) were purchased from Watanabe Chemical Industries, Ltd. (Hiroshima, Japan) and used as received. The other chemicals were purchased from FUJIFILM Wako Pure Chemical Industries, Ltd. (Osaka, Japan), or Tokyo Chemical Industry Co., Ltd. (Tokyo, Japan) and were used as received without purification unless otherwise noted.

**Synthesis of diamine initiators sandwiched by glycine (G1-G4)**

*N*-Boc-glycine (40 mmol, 7.00 g), OxymaPure (40 mmol, 5.68 g), 1,3-phenylenediamine (Pda, 20 mmol, 2.16 g) and triethylamine (TEA, 35 mL) in dichloromethane (DCM, 40 mL) were added to a flask equipped with an addition funnel and stir bar at -10 °C under nitrogen. A solution of EDC∙HCl (40 mmol, 7.67 g) in DCM (40 mL) was added dropwise over 30 min, and the resulting mixture was stirred at −10 °C for 30 min and then at 25 °C for 24 h. The mixture was subsequently washed with 4 wt% KHSO_4_. (3×100 mL), sat. NaHCO_3_ aq. (3×100 mL) and saltwater. The organic layer was dried with Na_2_SO_4_ and concentrated via a rotary evaporator. The product was dried in vacuo to give Boc-GlyPdaGly Boc as a pale-brown solid. The obtained Boc-GlyPdaGly Boc was then subjected to deprotection of the Boc group. The crude product was then dissolved in DCM (40 mL), and trifluoroacetic acid (TFA, 40 mL) was added to the solution. The mixture was stirred at 25 °C for 2 h. After the solvent was removed under reduced pressure, the crude product was dissolved in dioxane/HCl (4.0 M, 5 mL). The solution was poured into di-isopropyl ether, and the precipitate was filtered, washed with di-isopropyl ether, and dried under vacuum to yield GlyPdaGly∙2HCl (**G1**) as a pale yellow solid. The yield was 4.58 g (77.7%). The chemical structure of the product was characterized by ^1^H NMR spectroscopy and ESI‒MS. All other diamine-type tripeptide monomers (**G2**, **G3** and **G4**) were prepared via the same experimental procedure.

GlyPdaGly∙2HCl (**G1**)

ESI-MS (*m/z*): [M+Na]^+^ Calcd for C_10_H_14_N_4_O_2_Na, 245.1009; found, 245.1015.

^1^H NMR (DMSO-*d*_6_, 400 MHz, δ ppm): 8.24 (s, 6H), 7.94 (s, 1H), 7.38-7.34 (d, 2H), 7.31-7.27 (q, 1H), 3.79-3.77 (d, 4H).

GlyDanGly∙2HCl (**G2**)

ESI-MS (*m/z*): [M+Na]^+^ Calcd for C_11_H_16_N_4_O_3_Na, 275.1115; found, 275.1117.

^1^H NMR (DMSO-*d*_6_, 400 MHz, δ ppm): 8.24 (s, 6H), 7.44-7.42 (d, 1H), 7.14-7.11 (d, 1H), 7.06 (d, 1H), 3.84-3.82 (d, 4H).

GlyDapGly∙2HCl (**G3**)

ESI-MS (*m/z*): [M+Na]^+^ Calcd for C_10_H_14_N_4_O_3_Na, 261.0958; found, 261.0956.

^1^H NMR (DMSO-*d*_6_, 400 MHz, δ ppm): 10.42 (s, 1H), 9.77 (s, 2H), 8.20 (s, 4H), 7.27-7.24 (d, 1H), 7.04-7.02 (d, *J* = 8.0 Hz, 1H), 6.89-6.87 (d, 1H), 3.84-3.82 (d, 4H).

GlyDarGly∙2HCl (**G4**)

ESI-MS (*m/z*): [M+Na]^+^ Calcd for C_10_H_14_N_4_O_4_Na, 277.0907; found, 277.0908.

^1^H NMR (DMSO-*d*_6_, 400 MHz, δ ppm): 8.48 (s, 2H), 8.29 (s, 4H), 8.17 (s, 2H), 7.91 (s, 1H), 6.58 (s, 1H), 3.80-3.78 (d, 4H).

**Synthesis of diamine initiators sandwiched by alanine (A1-A4)**

*N*-Boc-alanine (40 mmol, 7.57 g), OxymaPure (40 mmol, 5.68 g), 1,3-phenylenediamine (Pda, 20 mmol, 2.16 g) and triethylamine (TEA, 35 mL) in dichloromethane (DCM, 40 mL) were added to a flask equipped with an addition funnel and stir bar at −10 °C under nitrogen. A solution of EDC∙HCl (40 mmol, 7.67 g) in DCM (40 mL) was added dropwise over 30 min, and the resulting mixture was stirred at −10 °C for 30 min and then at 25 °C for 24 h. The mixture was subsequently washed with 4 wt% KHSO_4_. (3×100 mL), sat. NaHCO_3_ aq. (3×100 mL) and saltwater. The organic layer was dried with Na_2_SO_4_ and concentrated via a rotary evaporator. The product was dried in vacuo to give Boc-AlaPdaAla-Boc as a pale-brown solid. The obtained Boc-AlaPdaAla-Boc was then subjected to deprotection of the Boc group. The crude product was then dissolved in DCM (40 mL), and trifluoroacetic acid (TFA, 40 mL) was added to the solution. The mixture was stirred at 25 °C for 2 h. After the solvent was removed under reduced pressure, the crude product was dissolved in dioxane/HCl (4.0 M, 5 mL). The solution was poured into di-isopropyl ether, and the precipitate was filtered, washed with di-isopropyl ether, and dried under vacuum to yield AlaPdaAla∙2HCl (**A1**) as a pale yellow solid. The yield was 6.01 g (93.0%). The chemical structure of the product was characterized by ^1^H NMR spectroscopy and ESI‒MS. All other diamine-type tripeptide monomers (**A2**, **A3** and **A4**) were prepared via the same experimental procedure.

AlaPdaAla∙2HCl (**A1**)

ESI-MS (*m/z*): [M+Na]^+^ Calcd for C_12_H_18_N_4_O_2_Na, 273.1322; found, 273.1320.

^1^H NMR (DMSO-*d*_6_, 400 MHz, δ ppm): 8.31 (s, 6H), 7.60 (s, 1H), 7.40-7.36 (d, 2H), 7.34-7.28 (q, 1H), 4.07 (q, 2H), 1.46-1.44 (d, 6H).

AlaDanAla∙2HCl (**A2**)

ESI-MS (*m/z*): [M+Na]^+^ Calcd for C_13_H_20_N_4_O_3_Na, 303.1428; found, 303.1428.

^1^H NMR (DMSO-*d*_6_, 400 MHz, δ ppm): 8.33 (s, 6H), 7.65 (s, 1H), 7.44 (d, 1H), 7.12-7.10 (d, 1H), 4.05-4.03 (q, 2H), 3.84 (s, 2H), 1.46-1.44 (d, 6H).

AlaDapAla∙2HCl (**A3**)

ESI-MS (*m/z*): [M+Na]^+^ Calcd for C_12_H_18_N_4_O_3_Na, 289.1271; found, 289.1273.

^1^H NMR (DMSO-*d*_6_, 400 MHz, δ ppm): 10.55 (s, 1H), 8.37 (s, 6H), 8.08-8.07 (d, 1H), 7.29-7.26 (q, 1H), 6.91-6.89 (d, 1H), 4.01-4.03 (q, 2H), 1.44-1.42 (d, 6H).

AlaDarAla∙2HCl (**A4**)

ESI-MS (*m/z*): [M+Na]^+^ Calcd for C_12_H_18_N_4_O_4_Na, 305.1220; found, 305.1219.

^1^H NMR (DMSO-*d*_6_, 400 MHz, δ ppm): 8.48 (s, 2H), 8.29 (s, 4H), 8.17 (s, 2H), 7.91 (s, 1H), 6.58 (s, 1H), 4.09-4.07 (d, 2H), 1.44-1.39 (m, 6H).

**Characterization procedures**

^1^H NMR spectra were recorded on a Bruker DPX400 spectrometer (Bruker, Bremen, Germany) at 400 MHz. Deuterated dimethylsulfoxide (DMSO-*d*_6_) and deuterated trifluoroacetic acid (TFA-*d*) were used as the solvents, and tetramethylsilane (TMS) served as an internal standard for the polypeptides. MALDI-TOF MS spectra were recorded with an AutoFlex III Plus (Bruker) spectrometer using α-cyano-4-hydroxycinnamic acid (α-CHCA) as the matrix dissolved in acetonitrile containing 0.1% TFA.

The Fourier transform infrared (FT-IR) spectra of the collected precipitate samples were recorded by using an IR Prestige-21 Fourier transform infrared spectrophotometer (Shimadzu Corporation, Kyoto, Japan) with a MIRacle A single-reflection attenuated total reflection unit using a Ge prism.

**Measurement procedures**

Thermogravimetric analysis (TGA) was performed on the polymer samples via TGA/DSC2 (Mettler Toledo, Columbus, OH, USA). The polymer sample (~5 mg) was weighed on an aluminum pan and heated with an empty reference cell at a heating rate of 20 °C min^−1^ from 30 to 500 °C under nitrogen. Differential scanning calorimetry (DSC) measurements were performed on the polypeptide samples via a DSC 8500 (PerkinElmer, Waltham, MA, USA). The polypeptide sample (~5 mg) was weighed on an aluminum pan and subjected to heating/cooling cycles at a heating rate of 20 °C min^−1^ and a cooling rate of 100 °C min^−1^ over a range from -50 to 250 °C under nitrogen.

**Wide-angle X-ray diffraction (WAXD) measurements**

The synchrotron WAXD measurements of the polypeptide powdery samples were performed on the BL05XU beamline (SPring-8, Harima, Japan) using an X-ray energy of 12.4 keV (wavelength: 0.1 nm); a beam with a diameter of 45 μm was employed. The obtained two-dimensional (2D) diffraction patterns were converted to one-dimensional (1D) profiles via azimuthal integration via Fit2D.

**Observation of polypeptide crystals via atomic force microscopy (AFM)**

Polypeptides obtained from the chemoenzymatic polymerization were dispersed in hexafluoro-2-propanol (HFIP) and methanol (1:1 v/v, 1 mg mL^–1^) by sonication for 30 min at room temperature. After the dispersion was incubated at room temperature for 24 h, an aliquot (5 μL) of the supernatant was deposited on a mica substrate and then dried at room temperature for more than 12 h. The samples were subjected to AFM observation in both topographic and phase modes. The AFM observations were performed on an AFM100 Plus (Hitachi High-Tech Science Corporation, Tokyo, Japan) in dynamic force mode (topographic and phase modes) with an SI-DF-40P2 cantilever (resonant frequency: 300 kHz, force constant: 26.0 N m^–1^) for the samples on the mica substrate.

**Table S1.** Full width at half maximum (FWHM) values for the peaks at 15.1 nm^-1^ in the WAXD patterns of TPGs

| Polymer | FWHM of 15.1 nm^-1^ (nm^-1^) |
| --- | --- |
| PolyGly | 0.243 |
| TPG1 | 0.253 |
| TPG2 | 0.400 |
| TPG3 | 0.397 |
| TPG4 | 0.443 |

**Table S2.** Full width at half maximum (FWHM) values for the peak at 11.9 nm^-1^ in the WAXD patterns of the TPAs

| Polymer | FWHM of 11.9 nm^-1^ (nm^-1^) |
| --- | --- |
| PolyAla | 0.549 |
| TPA1 | 0.581 |
| TPA2 | 0.531 |
| TPA3 | 0.856 |
| TPA4 | 0.768 |


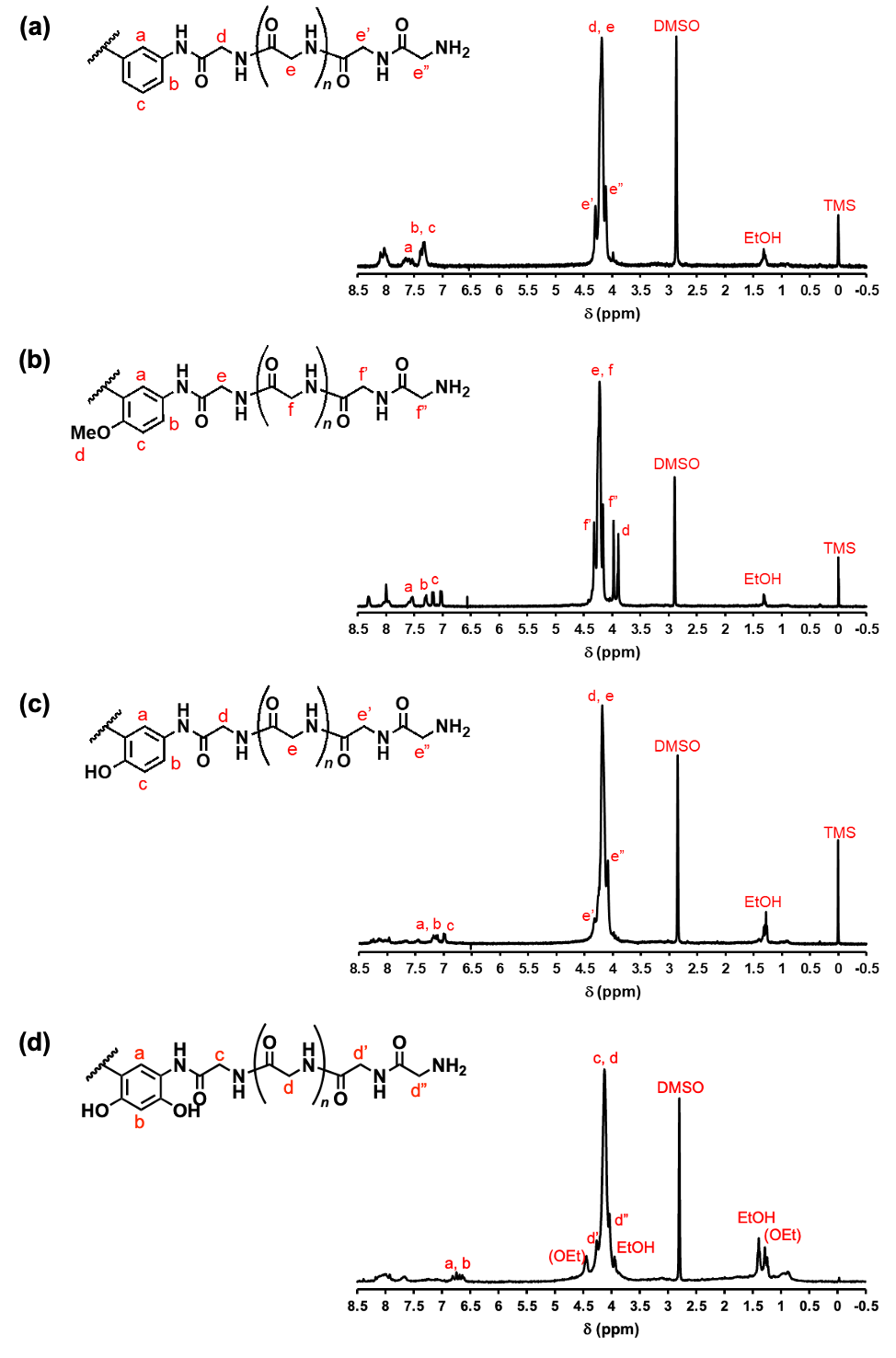


**Figure S1.** ^1^H NMR spectra of **TPG1-4** (solvent: DMSO-*d*_6_/TFA-*d* (1:5 v/v))


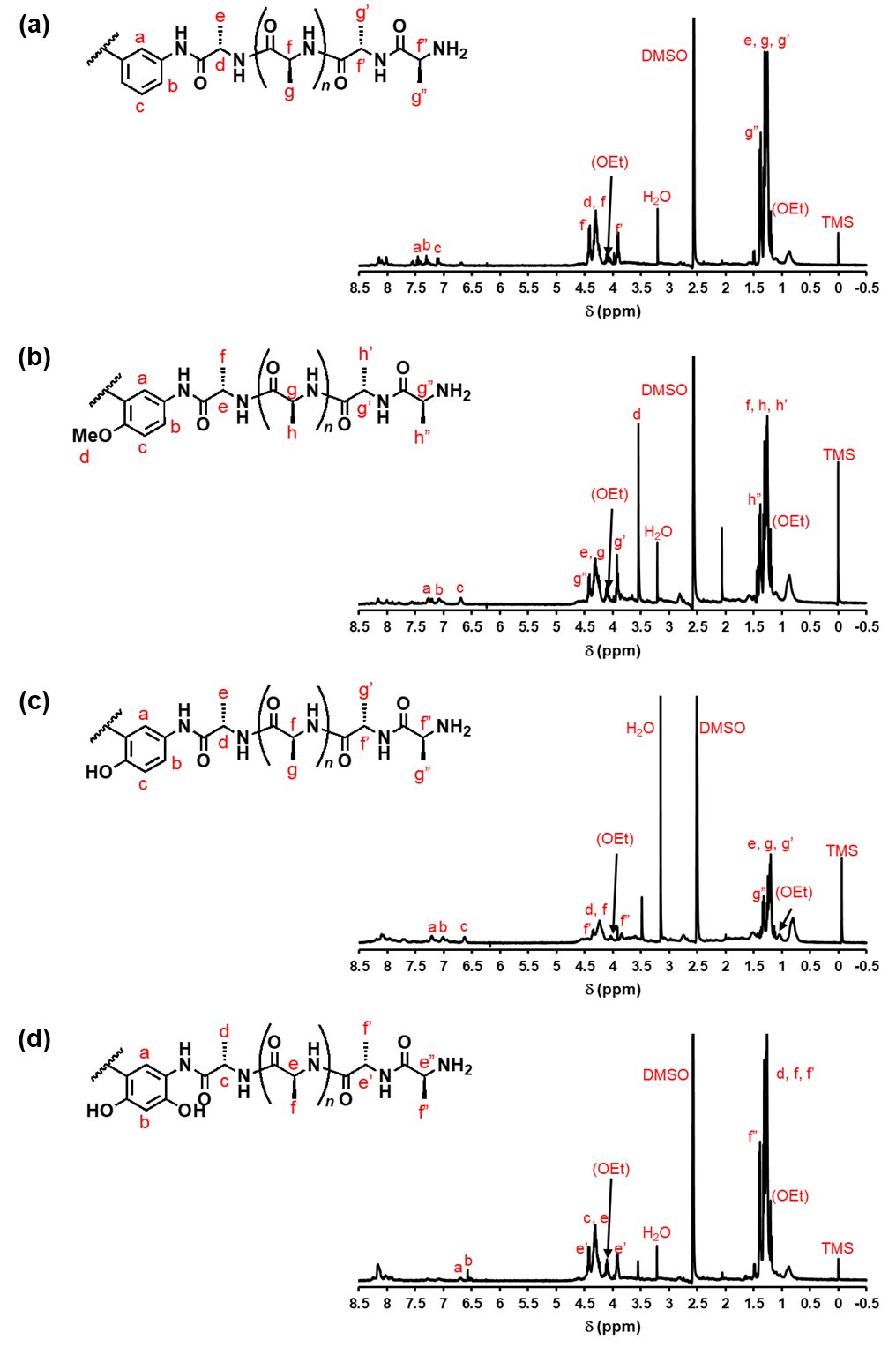


**Figure S2.** ^1^H NMR spectra of **TPA1-4** (solvent: DMSO-*d*_6_/TFA-*d* (5:1 v/v))


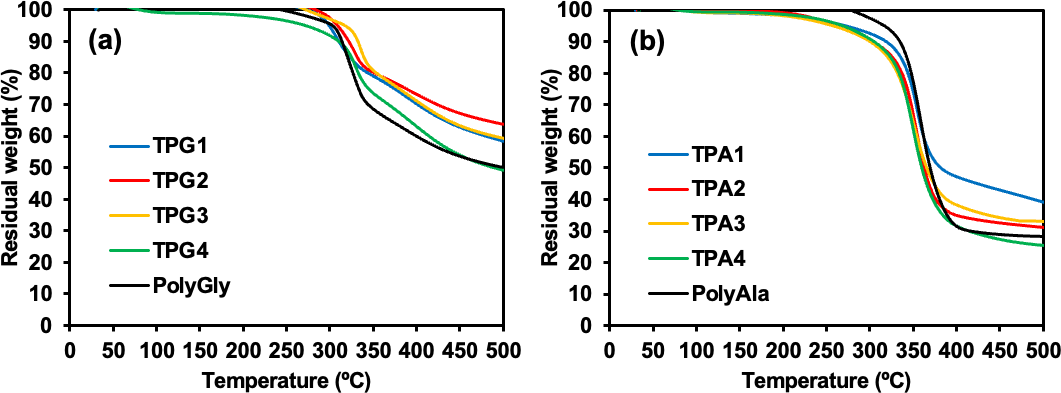


**Figure S3.** TGA curves (20 °C min−1 for heating) of (a) **TPG1-4** and PolyGly and (b) **TPA1-4** and PolyAla.


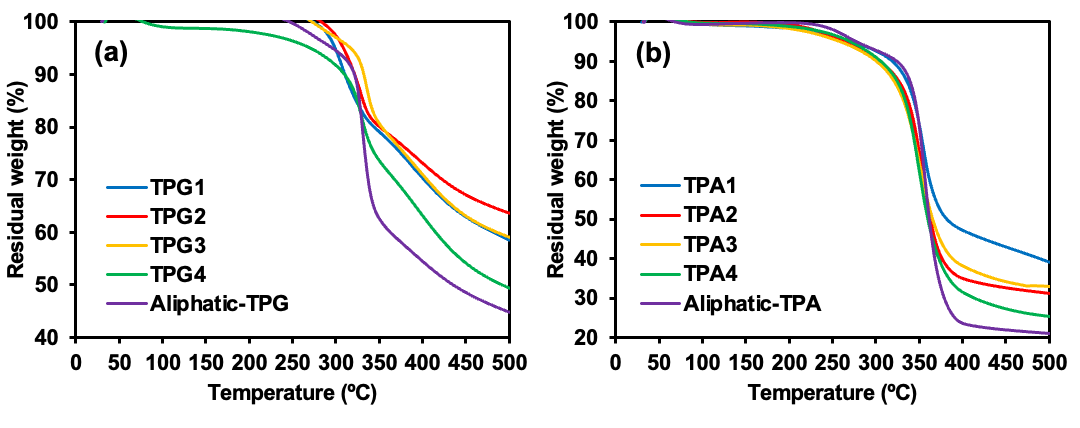


**Figure S4.** TGA curves (20 °C min^−1^ for heating) of (a) **TPG1-4** and aliphatic-TPG and (b) **TPA1-4** and aliphatic-TPA


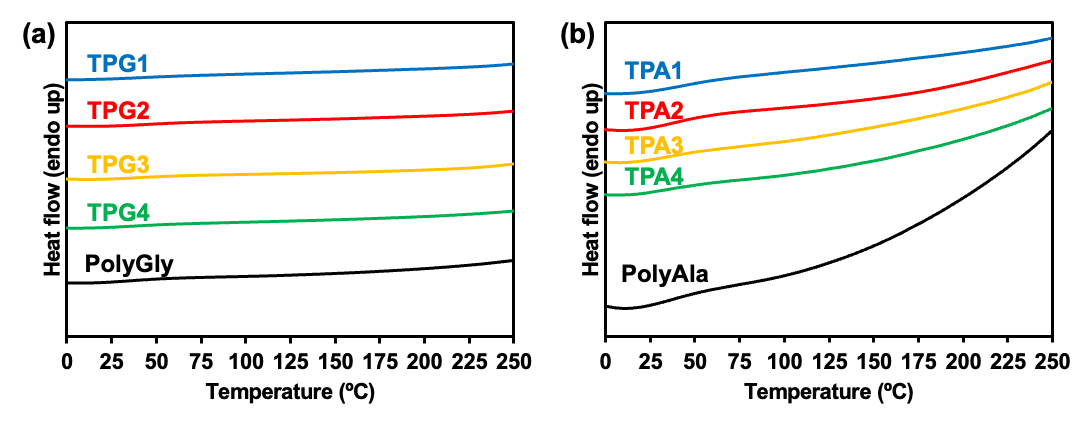


**Figure S5.** DSC profiles (20 °C min^−1^ for heating) of (a) **TPG1-4** and PolyGly and (b) **TPA1-4** and PolyAla
