# Supplementary figures and images for: Synthesis of Telechelic‐Type Polypeptides Functionalized with Aromatic Units and the Characterization of Their Structures and Thermal Properties
Source: Macromol Rapid Commun. 2025 Aug 26;47(10):e00499. doi: 10.1002/marc.202500499 (PMC13193375; doi:10.1002/marc.202500499)

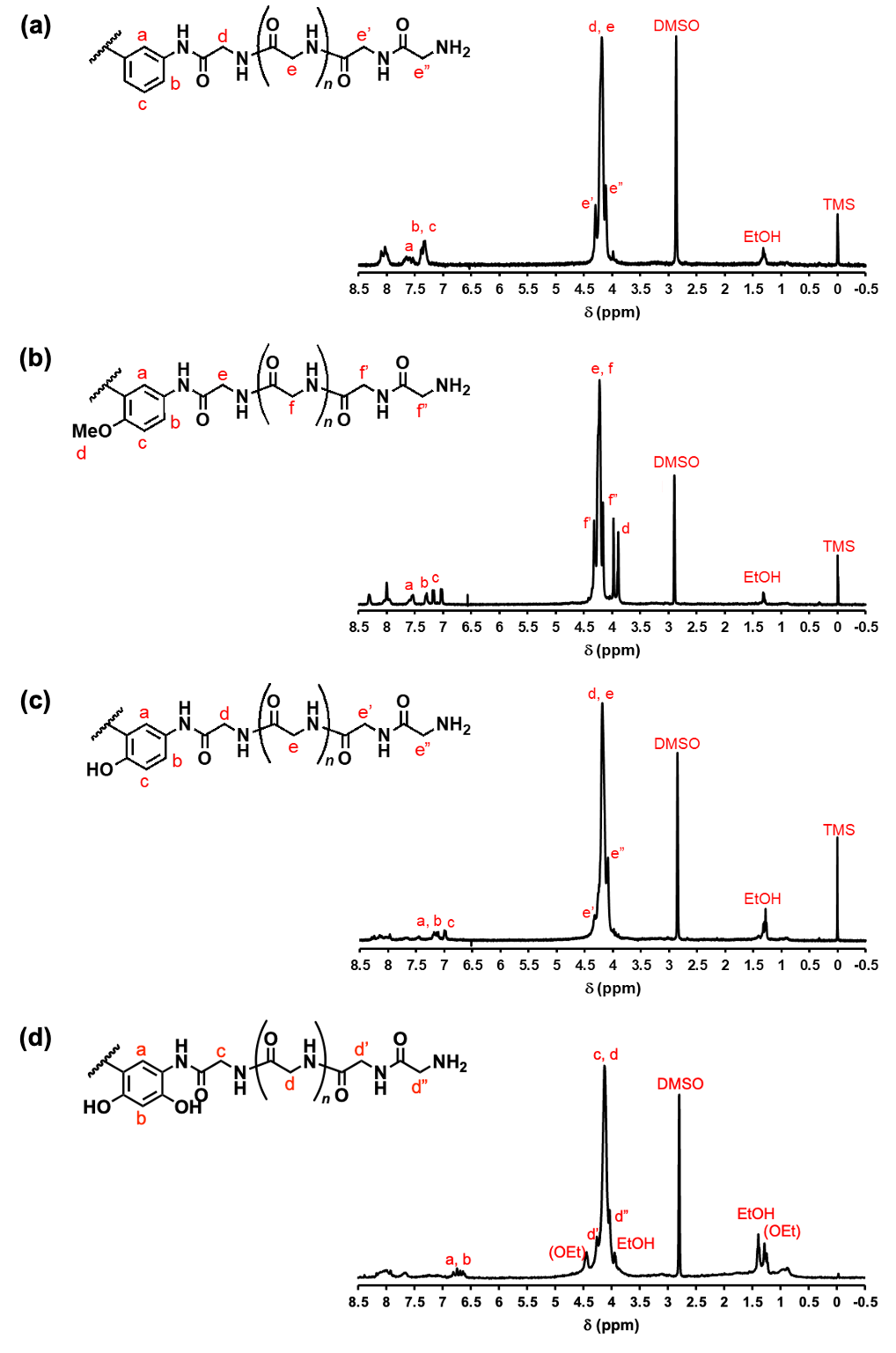

Supplement: Supplementary file 2 — Supporting File 2: marc70045‐sup‐0002‐FigureS1.png. [file MARC-47-e00499-s006.png]

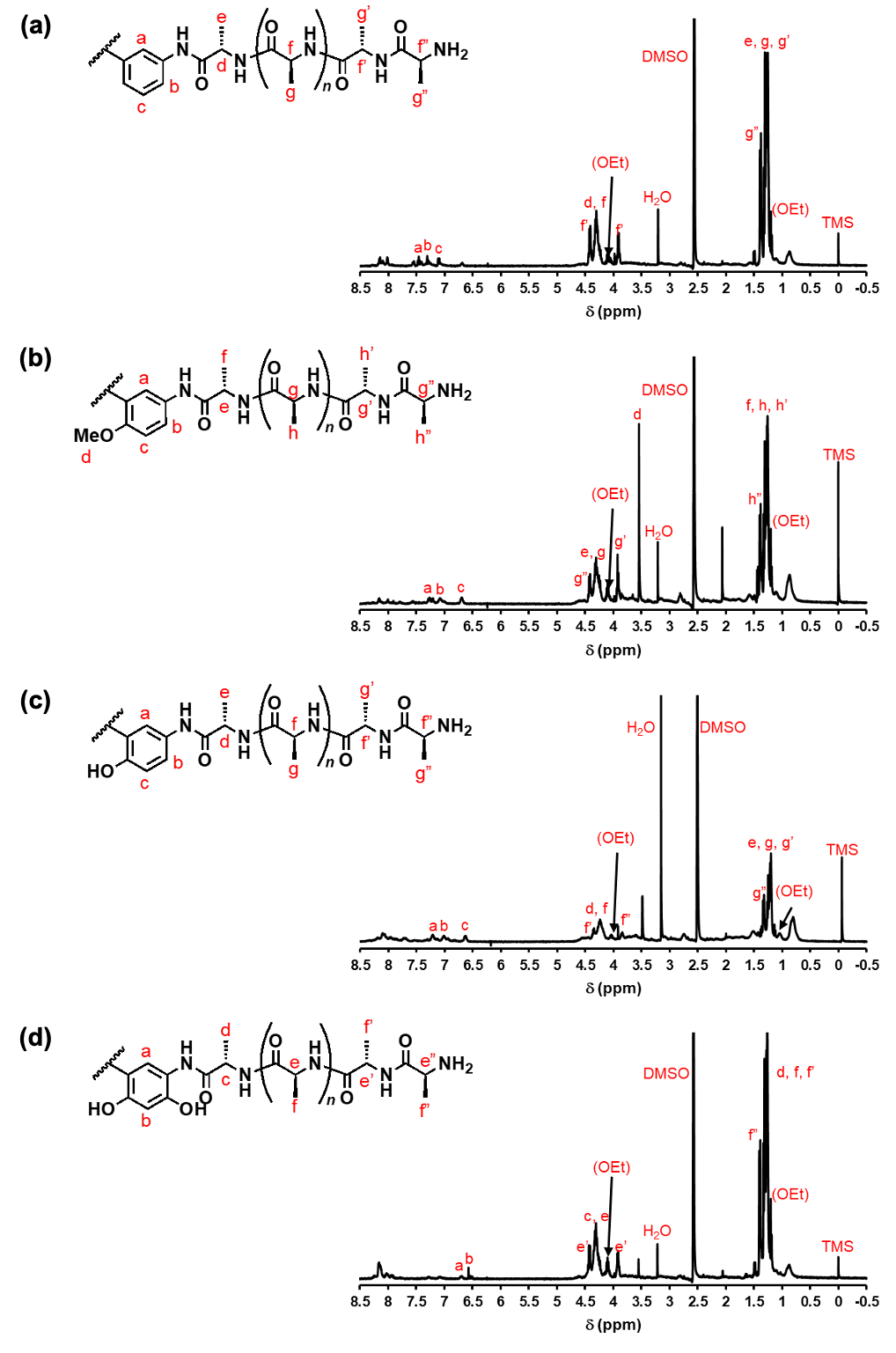

Supplement: Supplementary file 3 — Supporting File 3: marc70045‐sup‐0003‐FigureS2.png. [file MARC-47-e00499-s002.png]

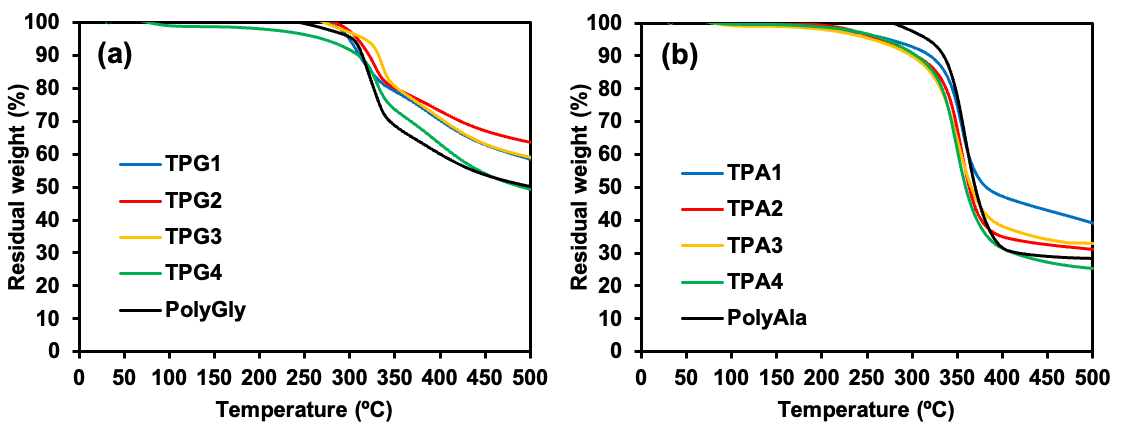

Supplement: Supplementary file 4 — Supporting File 4: marc70045‐sup‐0004‐FigureS3.png. [file MARC-47-e00499-s004.png]

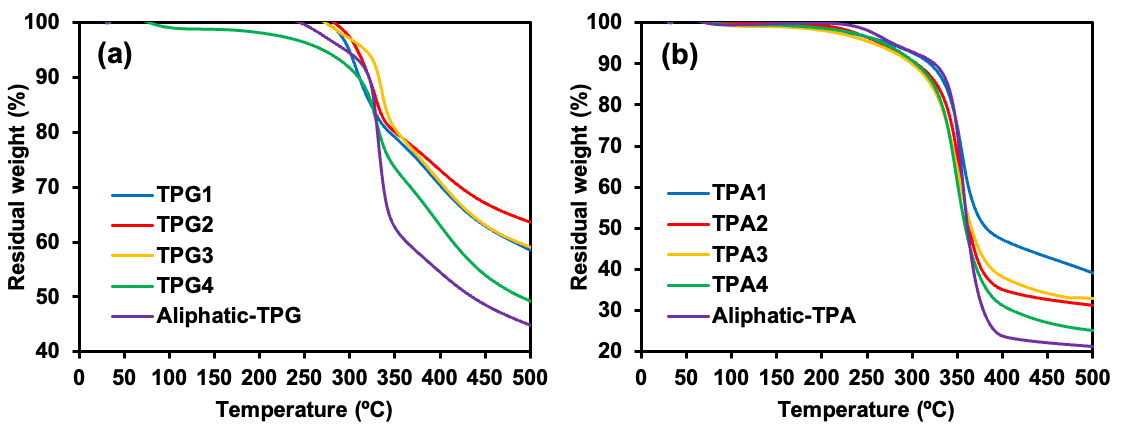

Supplement: Supplementary file 5 — Supporting File 5: marc70045‐sup‐0005‐FigureS4.png. [file MARC-47-e00499-s003.png]

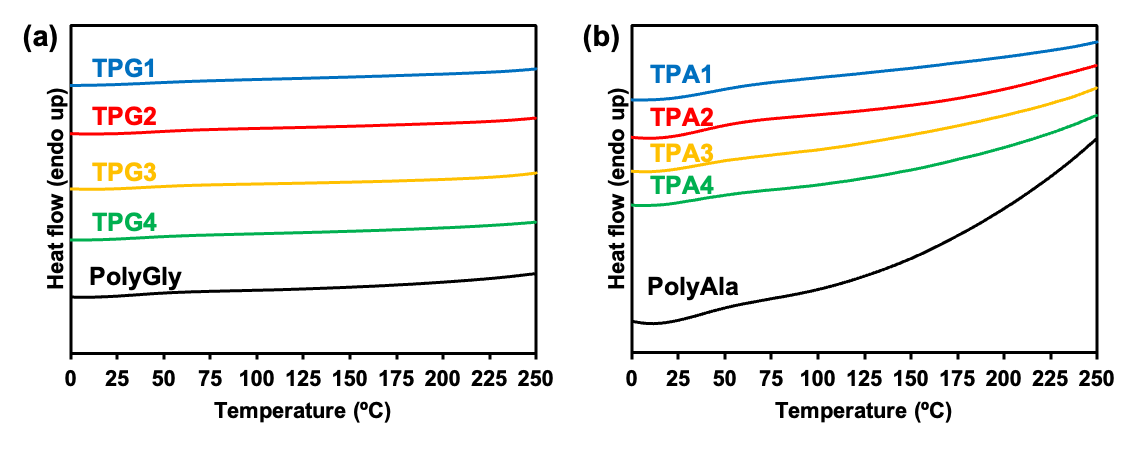

Supplement: Supplementary file 6 — Supporting File 6: marc70045‐sup‐0006‐FigureS5.png. [file MARC-47-e00499-s001.png]
